# An In Vitro Cell Model of Intestinal Barrier Function Using a Low-Cost 3D-Printed Transwell Device and Paper-Based Cell Membrane

**DOI:** 10.3390/ijms26062524

**Published:** 2025-03-12

**Authors:** Pitaksit Supjaroen, Wisanu Niamsi, Parichut Thummarati, Wanida Laiwattanapaisal

**Affiliations:** 1 Graduate Program in Clinical Biochemistry and Molecular Medicine, Department of Clinical Chemistry, Faculty of Allied Health Sciences, Chulalongkorn University, Bangkok 10330, Thailand; pitaksit.sa@gmail.com (P.S.);; 2 Centre of Excellence for Biosensors and Bioengineering (CEBB), Department of Clinical Chemistry, Faculty of Allied Health Sciences, Chulalongkorn University, Bangkok 10330, Thailand; parichut.t@chula.ac.th; 3 Department of Clinical Chemistry, Faculty of Allied Health Sciences, Chulalongkorn University, Bangkok 10330, Thailand

**Keywords:** intestinal barrier, neutrophil extracellular trap (NET), paper-based membrane, 3D-printed transwell device, transepithelial electrical resistance (TEER), permeability assay

## Abstract

Current in vitro methods for intestinal barrier assessment predominantly utilize two-dimensional (2D) membrane inserts in standard culture plates, which are widely recognized for their inability to replicate the microenvironment critical to intestinal barrier functionality. Our study focuses on creating an alternative method for intestinal barrier function by integrating a 3D-printed transwell device with a paper-based membrane. Caco-2 cells were grown on a Matrigel-modified paper membrane, in which the tight junction formation was evaluated using TEER measurements. Neutrophil-like dHL-60 cells were employed for neutrophil extracellular trap (NET) formation experiments. Furthermore, intestinal barrier dysfunction was demonstrated using NET-isolated and Staurosporine interventions. Intestinal barrier characteristics were investigated through immunofluorescence staining of specific proteins and scanning electron microscopy (SEM). Our paper-based intestinal barrier exhibited an increased resistance in a time-dependent manner, consistent with immunofluorescence images of Zonulin Occludens-1 (ZO-1) expression. Interestingly, immunofluorescence analysis revealed changes in the morphology of the intestinal barrier and the formation of surface villi. These disruptions were found to alter the localization of tight junctions, impacting epithelial polarization and surface functionality. Moreover, we successfully demonstrated the permeability of a paper-based intestinal barrier using FITC-dextran assay. Hence, the 3D-printed transwell device integrated with a paper membrane insert presents a straightforward, cost-effective, and sustainable platform for an in vitro cell model to evaluate intestinal barrier function.

## 1. Introduction

The intestinal barrier is a crucial mechanical property of the small intestine to prevent and protect the intestine from harmful factors, serving as the primary interface between the body and the external environment. Understanding the processes that maintain the integrity of the intestinal barrier could lead to the development and discovery of alternative therapeutic approaches. The actual environment of the human intestine interacts with other compartments that act as an immunogenic barrier [1]. One of the predominant components of intestinal epithelial cells (IECs) is typically formed and polarized on a basement membrane. This arrangement can separate the intestinal compartment into the intestinal lumen and lamina propria [2]. The epithelial layer is a single-cell alignment that consists of different types of specialized differentiated cells on the connective tissue of a lamina propria compartment [3,4]. The intestinal barrier is typically cellular bound because of tight junction (TJ) protein complexes. Zonulin Occludens-1 (ZO-1) protein is a member of tight junctional proteins that play a role in the intestinal barrier function and regulate paracellular transportation and the diffusion of small molecules between cells [5,6]. Impairment of intestinal barrier function is associated with various conditions, such as neurodegenerative disorders, cardiovascular diseases, and functional gastrointestinal problems [7,8].

Several biological factors can influence and trigger intestinal inflammation, resulting in intestinal barrier dysfunction. Unfortunately, inadequate immune clearance and excessive immune system activation can induce prolonged or severe infection, revealing the immune-mediated condition of human diseases such as intestinal inflammatory bowel disease (IBD) [9]. As the first line of immune cells, neutrophils play a role in the host’s defense mechanism, preventing pathogenic invasion and alerting other cells by releasing cytokines and chemokines. By forming a neutrophil extracellular trap (NET), neutrophils can release several types of molecules to trap and kill the invasive pathogen. NET comprises an extracellular network of decondensed chromatin embedded with microbicidal proteins and oxidative enzymes, such as myeloperoxidase (MPO), neutrophil elastase (NE), and filamentous-like DNA [1,10]. Accordingly, decondensed DNA fiber is a long filamentous extracellular web-like structure activated through the NOX-dependent lytic NET formation by phorbol-12-myristate-13-acetate (PMA) [11]. This agonist is commonly used to stimulate NET formation by activating protein kinase C (PKC) through the toll-like receptor (TLR), followed by inducing the production of reactive oxygen species (ROS), which is a hallmark of PMA-stimulated NET formation [12,13]. Consequently, NETs promote the breakdown of intestinal barrier formation by inducing intestinal cell apoptosis and altering the integrity of tight junction proteins [1]. Therefore, the targeting approaches of NET inhibition provide a promising therapeutic strategy to alleviate intestinal inflammation and improve barrier integrity.

According to the in vitro assessment of intestinal barrier function, it can be determined using both in vitro and in vivo techniques. The in vitro method is a common way to access the gut barrier by culturing the intestinal epithelial cells on a porous membrane insert that allows two-separated systems of apical and basolateral compartments [14]. Meanwhile, a porous membrane of the commercial 2D transwell insert promotes cell growth in a two-dimensional (2D) system. Generally, a 2D cell culture system is known to lack the ability to mimic the microenvironment required for proper intestinal barrier function. Developing a platform to improve the biochemical cues in a three-dimensional (3D) cell culture system is challenging for researchers. In addition, a co-culture system was used to model an advanced intestinal barrier with different cell types [15,16]. Further integration of 3D cell culture models with microfluidic chips shows promise for advanced biomedical applications [17]. Interestingly, a scaffold membrane was exploited to mimic the basement membrane of the lamina propria compartment, which can promote cell growth, proliferation, and differentiation. Utilizing the scaffold membrane is flexible and depends on its physical properties, such as porosity, thickness, and membrane sources. Several sources of biocompatible materials can be used to fabricate the scaffold membrane for a culture environment. Accordingly, a simple, low-cost, and commonly available material in general laboratories, Whatman filter paper is promising for alternative substrates. It comprises cellulose microfiber, which has several beneficial purposes in various biomedical applications, such as a 3D scaffold for cell culture, a microfluidic device platform, and paper-based cell cryopreservation [18,19,20]. In this work, we have presented a 3D scaffold of a paper membrane to model the intestinal barrier and assess its function by measuring transepithelial electrical resistance (TEER) and permeability assay, verifying their characteristics using immunofluorescence staining. These techniques have been widely used to assess intestinal barrier function [9,21,22].

Wax or inkjet printing is a simple fabrication technique to define and pattern the hydrophilic functional area on the paper membrane [19,23]. Subsequently, the membrane can be further modified with an extracellular matrix (ECM) enriched with ECM proteins, such as laminin, collagen IV, and entactin [24], providing an appropriate microenvironment for culturing cells. A Matrigel-functionalized membrane for a 3D cell culture system of in vitro cell modeling is one example [25]. Moreover, our previous research successfully fabricated a simple and versatile 3D-printing device to culture intestinal epithelial cells on a paper membrane [26,27]. Thus, 3D printing technology offers several advantages in therapeutic approaches and research applications. It is a flexible, customizable, and cost-effective solution for fabricating biocompatible devices used in the treatment of intestinal diseases, cell culture platforms, and cell-based biosensors [28,29,30,31]. Additionally, various types of biocompatible filaments can be selected based on the intended applications. For instance, polylactic acid (PLA) filament is a well-known biocompatible material that offers an environmentally friendly option and demonstrates good compatibility for cell culture applications and the fabrication of biomedical devices [32,33,34].

This study aims to provide a low-cost, promising, in vitro cell model for intestinal barrier function using a customizable 3D-printed transwell device coupled with a paper membrane. Comprehensive investigations were conducted to confirm its applicability, including cell barrier characterization and monitoring changes following the intervention with biological and chemical stimuli. The results confirm that our proposed device offers a simple-to-handle platform employing different methods, including a standard method of TEER measurement, immunofluorescence analysis, and a permeability assay of FITC-dextran. Moreover, these findings provide a significant in vitro cell model for intestinal barrier function and will be beneficial for understanding biological mechanisms, tissue engineering, drug screening, and drug discovery.

## 2. Results

### 2.1. Assessment of a Paper-Based Intestinal Barrier Membrane

**Figure 1:**
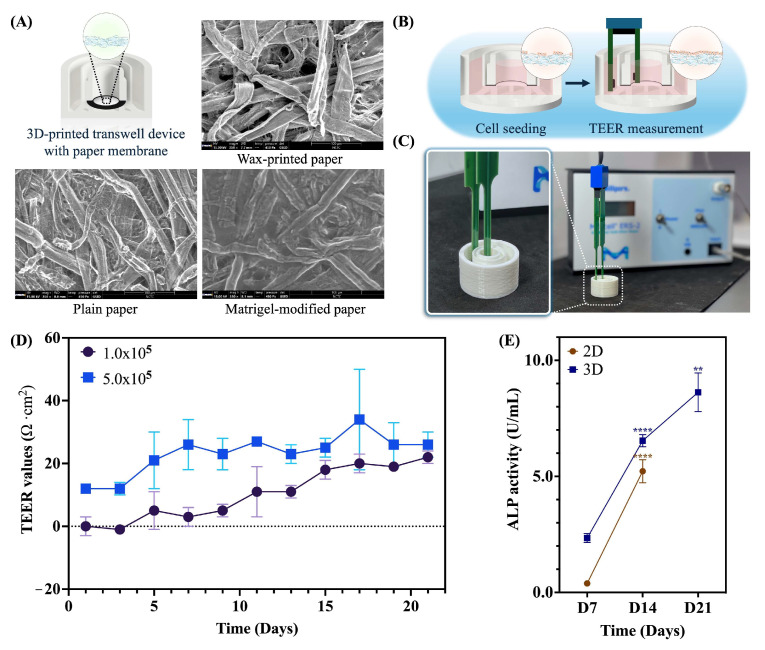
(**A**) SEM images of a paper characterization at three regions: wax-printed, plain, and Matrigel-modified areas. (**B**) The schematic illustration represents the experimental setup for the TEER application. (**C**) Photographs of a 3D-printed transwell device with a chopstick electrode setting. (**D**) TEER values of Caco-2 cells cultured on a Matrigel-modified paper from day 1 to day 21. (**E**) The ALP activity of Caco-2 cells on both 2D and 3D membranes. The results are presented as mean ± standard deviation (SD) (*n* = 3). Statistical analysis was performed using two-way ANOVA followed by Tukey’s test. Significant differences are indicated by ** *p* < 0.01 between the ALP activity of Day 14 and Day 21 in 3D culture and **** *p* < 0.0001 between the ALP activity of Day 7 and 14 in both 2D and 3D cultures. (Personal creation elaborated with Biorender.com).

The characteristics of the paper membrane, which acts as the basement membrane for intestinal barrier formation, are displayed in Figure 1A. SEM images revealed the cell-free surface morphology across three distinct regions, including wax-printed, plain, and Matrigel-modified areas. This finding suggested a smooth surface on the Matrigel-modified paper, designated as the culturing area for Caco-2 cells, in contrast to the rough texture and microfiber network observed on the plain membrane. Meanwhile, the characteristics of commercial 2D membrane insert revealed a flatted porous membrane (Supplementary Figure S1A), which promotes cell growth in 2D morphological shapes and offers a higher resistance of TEER values than other alternative scaffold membranes (Supplementary Figure S1B). The TEER measurements of the 2D membrane insert revealed that the cells (1.0 × 10^5^ cells/cm^2^) peaked at 13 days of culture, followed by a decline in TEER resistance through to the 21-day culture, as shown in Supplementary Figure S1. This outcome suggests that the integrity of the cell barrier was compromised, resulting in increased permeability and indicating that the cells may not be suitable for future functional assays after 13 days of culture.

Furthermore, Caco-2 cells were cultured on a Matrigel-modified paper of a 3D-printed transwell device to establish a paper-based intestinal barrier model. The transepithelial electrical resistance of intestinal barrier formation was measured every second day using a chopstick electrode, as shown in Figure 1B,C. Interestingly, utilizing our device was detectable by a standard TEER electrode and exhibited an increased resistance signal in a time-dependent manner at both initial cell densities (Figure 1D). Additionally, an initial cell density of 1.0 × 10^5^ cells/cm^2^ exhibited a dynamically increased resistance in the range of 0 Ω·cm^2^ to 21 Ω·cm^2^, while an initial cell density of 5.0 × 10^5^ cells/cm^2^ exhibited a resistance in the range of 12 Ω·cm^2^ to 34 Ω·cm^2^ (Figure 1D). These findings indicated that the paper membrane was well-suited for supporting cell-to-cell interactions to establish barrier integrity.

After confirming the increased TEER values, the activity of alkaline phosphatase (ALP), a brush border enzyme used as a differentiation marker, was measured. In the 2D culture, the ALP activity on the apical side of the Caco-2 cells was initially low, measuring 0.39 ± 0.08 U/mL on day 7. However, this value significantly increased over time, reaching 5.22 ± 0.50 U/mL by day 14 (*p* < 0.0001), as shown in Figure 1E (represented by brown dots). Interestingly, the paper-based intestinal barrier membrane displayed a gradual increase, reaching 6.54 ± 0.26 U/mL by day 14 (*p* < 0.0001), as shown in Figure 1E (indicated by dark blue dots). By day 14, the ALP activity of Caco-2 cells on the paper membrane was 25% higher than that on the 2D membrane, suggesting enhanced differentiation and polarization of the Caco-2 cells.

### 2.2. Cell Characteristics of a Paper-Based Intestinal Barrier Membrane

**Figure 2:**
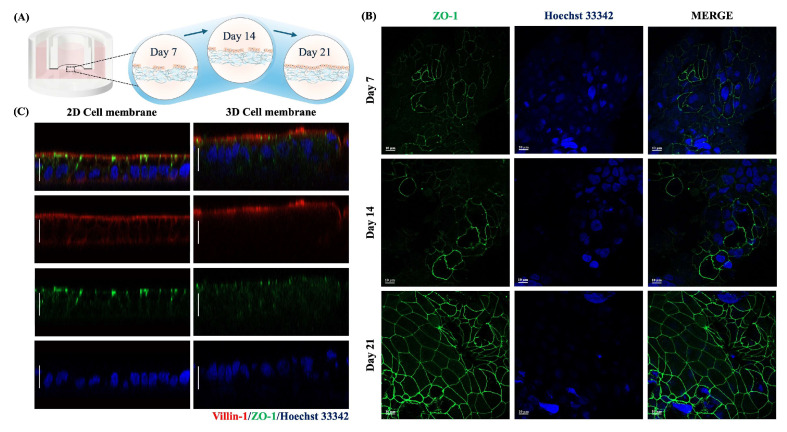
(**A**) Intestinal barrier formation using a 3D-printed transwell device coupled with a paper membrane. (**B**) Representative immunofluorescence images of ZO-1 protein expression on 7, 14, and 21-day-cultured Caco-2 cells. (**C**) Z-stack images of 2D and 3D cell membranes after culturing for 21 days. The scale bar is 10 µm. (Personal creation elaborated with Biorender.com).

The cell characteristics of the tight junction integrity and surface microvilli were determined by assessing the expression of the ZO-1 and villin-1 protein, respectively, on 7, 14, and 21-day-cultured Caco-2 cells (Figure 2A). As shown in Figure 2B, 7-day-cultured Caco-2 cells formed cell junctions with adjacent cells and revealed a greater expression of ZO-1 protein in a time-dependent manner, aligning with the TEER values. Notably, 21-day-cultured Caco-2 cells on a paper membrane demonstrated polarization by showing villin-1 localized on an apical compartment, resembling the results observed on a 2D cell membrane (Figure 2C). The tight junction integrity was also established about 10–15 µm in thickness on a paper-based membrane. These results suggested that Caco-2 cells differentiated into functional epithelial cells and interacted with neighbor cells in terms of cell-cell interaction.

**Figure 3:**
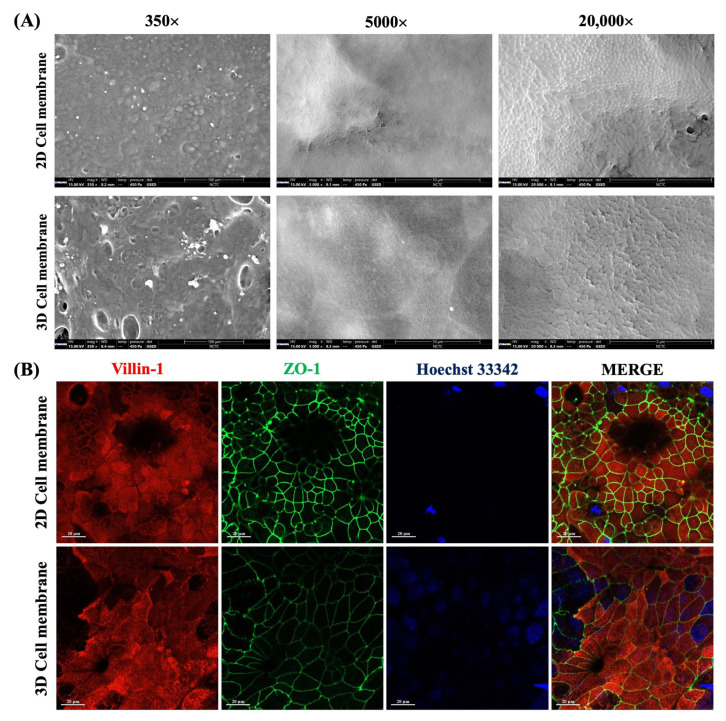
(**A**) The SEM images of 2D and 3D cell membranes of 21-day cultured Caco-2 cells at 350, 5000, and 20,000 magnifications. (**B**) Representative immunofluorescence images of specific proteins of 21-day-cultured Caco-2 on 2D and 3D cell membranes. The scale bar is 20 µm.

We further examined the surface morphology of both 2D and 3D cell membranes using SEM analysis. As shown in Figure 3A (350× magnification), Caco-2 cells were attached and spread to cover the culturing surface of Matrigel-modified paper. According to the microfiber structure of a paper membrane, the result showed that a 3D cell membrane revealed a more three-dimensional shape. In contrast, a 2D cell membrane displayed a flat morphology, consistent with previous Z-stack images (Figure 2C). In addition, Caco-2 cells revealed the microvilli structure on the apical surface (Figure 3B), indicating the cells were differentiated into functional epithelial cells. Notably, these findings suggest that we successfully developed a paper-based intestinal barrier membrane and, for the first time, demonstrated tight junction integrity using TEER measurements with our custom-made 3D-printed transwell device.

### 2.3. NET-Induced Intestinal Barrier Dysfunction

**Figure 4:**
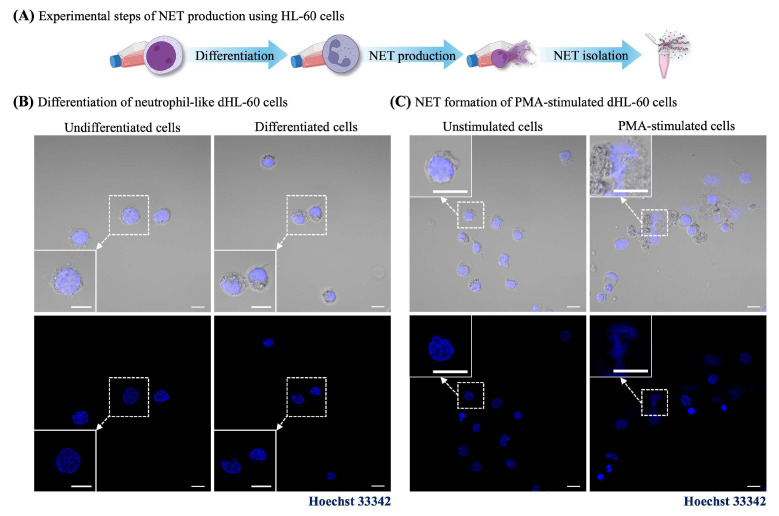
(**A**) Experimental steps of NET production. (**B**) Differentiation of neutrophil-like dHL-60 cells. (**C**) NET formation of PMA-stimulated dHL-60 cells using Hoechst 33342 staining. The images are shown in differential interference contrast (DIC) and immunofluorescence. The scale bar is 10 µm. (Personal creation elaborated with Biorender.com).

The intestinal barrier dysfunction caused by dsDNA from the NET process was investigated. The initial assessment of the physical morphology between undifferentiated and differentiated cells was observed under the light microscope, as shown in Supplementary Figure S2. Consistent with the fluorescence images of Hoechst 33342, staining showed that dHL-60 cells revealed the nuclear morphology in the lobular shape and reduced cell sizes to 10 μm, while undifferentiated HL-60 cells were approximately 15–20 μm, as shown in Figure 4B. Moreover, dHL-60 cells revealed good viability at day 6 post-differentiation (Supplementary Figure S3). This result indicated that dHL-60 cells were successfully differentiated into neutrophil-like cells. Consequently, the NET formation of dHL-60 cells through PMA induction was confirmed by Hoechst 33342 staining. The results showed that stained cells exhibited a lobular shape, decondensed, and released the DNA structure to the outside, showing several forms of NET-like structures (Figure 4C). Moreover, the NET-isolated supernatant was quantified for the dsDNA levels, and the results are shown in Supplementary Figure S4. This result indicates that PMA stimulation successfully induced the NET formation.

**Figure 5:**
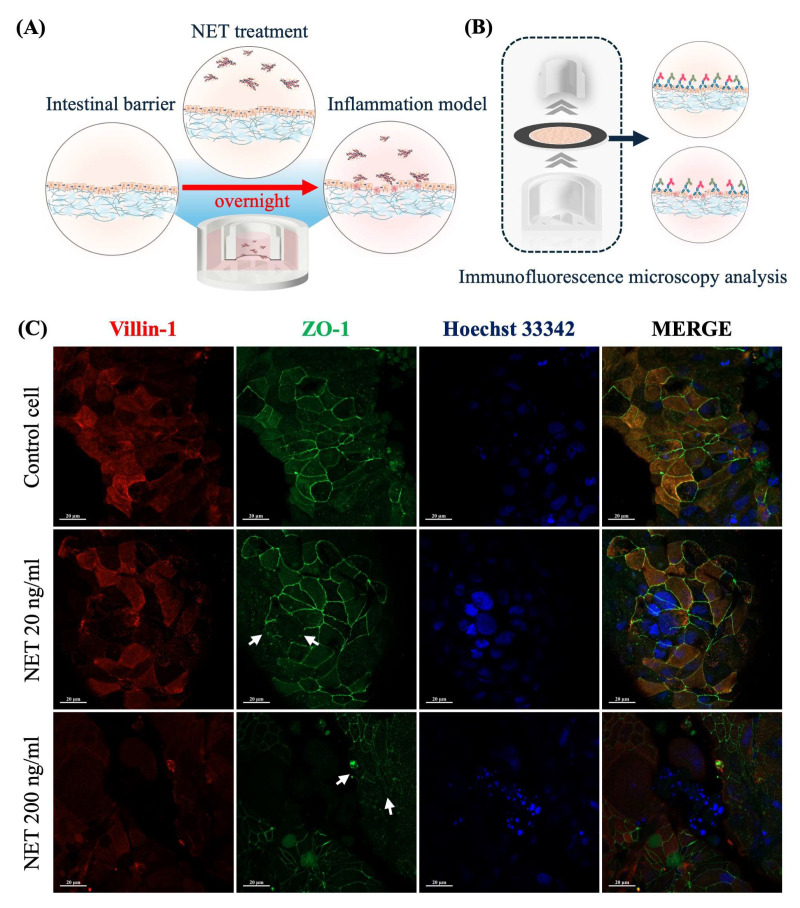
(**A**) Experimental conditions of a paper-based intestinal barrier at different interventions. (**B**) Deconstruction and preparing a paper membrane for fluorescence microscopy analysis. (**C**) Fluorescence images of cell characteristics (villin-1, ZO-1, and cell nuclei). White arrows highlight regions of disrupted ZO-1 localization, demonstrating a discontinuous tight junction. The scale bar is 20 μm. (Personal creation elaborated with Biorender.com).

Subsequently, intestinal barrier dysfunction through immune-mediated conditions using NET-isolated supernatant was demonstrated. Paper-based 21-day-cultured Caco-2 cells were treated with NET-isolated (dsDNA) supernatants at 20 ng/mL and 200 ng/mL in the culture medium overnight (Figure 5A). After completing the incubation period, the device was deconstructed and prepared for immunofluorescence assay (Figure 5B). Immunofluorescence images of a cell control group showed that the fluorescence signal of ZO-1 expression was predominantly localized in the cell boundaries, forming continuous and well-developed junctions. Additionally, the fluorescence signal of villin-1 expression was primarily localized at an apical compartment of polarized cells (Figure 5C). In contrast, the intervention of dsDNA induced a disruption of ZO-1 localization, showing a discontinuous pattern at the cell junctions, as indicated by the white arrows in Figure 5C. These results demonstrated that the NET-isolated dsDNA could directly disrupt tight junction integrity and alter the localization of surface microvilli.

### 2.4. Staurosporine-Induced Intestinal Barrier Dysfunction

**Figure 6:**
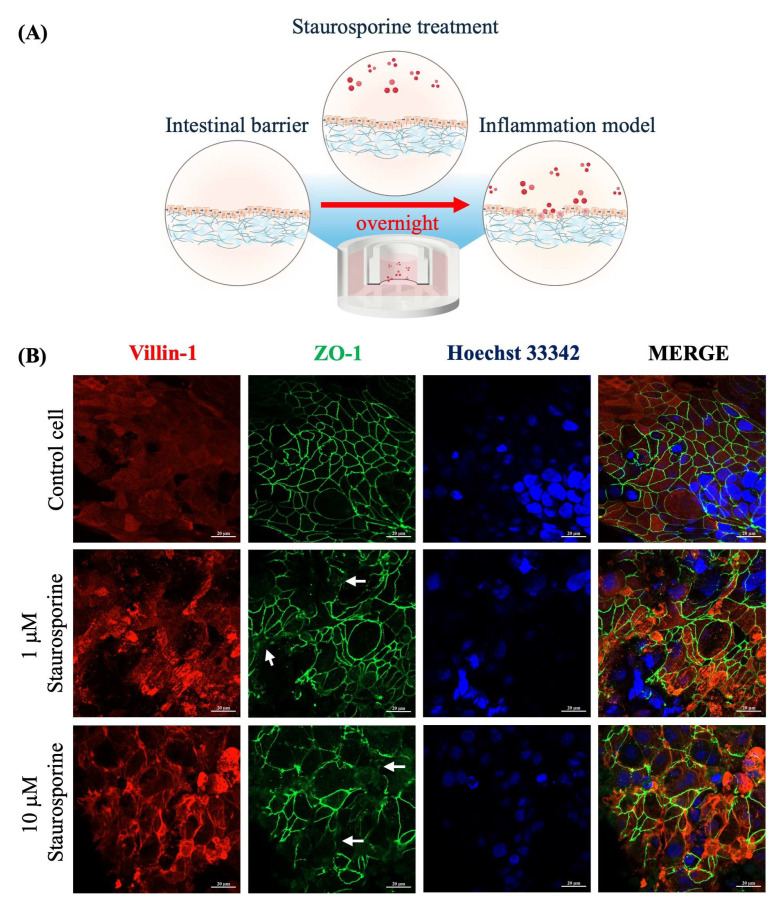
(**A**) Experimental conditions of intestinal inflammation model through Staurosporine interventions. (**B**) Representative fluorescence images of specific proteins of paper-based intestinal barrier membranes after Staurosporine incubation overnight. White arrows indicate regions of disrupted ZO-1 localization, highlighting a discontinuous tight junction and barrier dysfunction. The scale bar is 20 μm. (Personal creation elaborated with Biorender.com).

We further demonstrated the intestinal barrier dysfunction through Staurosporine induction, a chemical inducer of cell apoptosis [35]. After 21 days of culturing, Caco-2 cells were treated with Staurosporine overnight at 1 μM and 10 μM (Figure 6A). As demonstrated by immunofluorescence images, Caco-2 cells in a control group exhibited the fluorescence signal of ZO-1 expression at the cell boundaries, indicating a well-developed junction with adjacent cells. Additionally, the fluorescence signal of villin-1 expression was predominantly localized with distinct intensity at the apical compartment, indicating the well-formed microvilli (Figure 6B).

Conversely, Caco-2 cells treated with Staurosporine showed a dose-dependent disruption in the formation of ZO-1 protein, as highlighted by the white arrows in Figure 6B. Furthermore, Staurosporine could alter the localization of villin-1 protein (Figure 6B), suggesting a loss of microvilli structure. It can be implied that the Staurosporine could negatively affect the formation of tight junction protein and localization of surface microvilli.

This study investigated the cell viability of a paper-based intestinal barrier using LIVE/DEAD staining of Calcein-AM and PI solution. The working principle relies on dyes that differentially penetrate intact and compromised cell membranes. An increased uptake of the PI dye can indicate potential disruption of tight junction proteins, suggesting intestinal barrier dysfunction. As demonstrated by the fluorescence images in Supplementary Figure S5, the results show that NET and Staurosporine stimulations resulted in an intense red fluorescence signal, representing the localization of dead cells. In contrast, weak red fluorescence signals were observed in the control group.

### 2.5. Permeability Assay of a Paper-Based Intestinal Barrier Membrane

**Figure 7:**
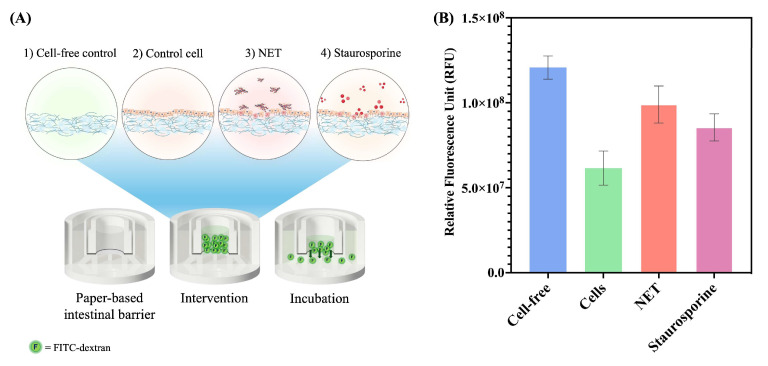
(**A**) The schematic illustration represents experimental conditions of the permeability assay employing FITC-dextran (4 kDa). (**B**) The permeability assay of paper-based intestinal barrier membranes post-intervention overnight. Results presented as mean ± standard deviation (SD) (*n* = 3). (Personal creation elaborated with Biorender.com).

The paper-based intestinal barrier equipped in a 3D-printed transwell device was employed for permeability assay based on the FITC-dextran assay (Figure 7A). Fascinatingly, the relative fluorescence unit (RFU) values in cell-free membranes were observed at the highest fluorescence intensity at 1.21 ± 0.07 × 10^8^ RFU. It could be indicated that the fluorescence tracer (FITC-dextran) can freely pass through the microfiber network of a Matrigel-modified paper membrane. Conversely, the membrane with cells exhibited approximately 49% decreased fluorescence intensity at 0.62 ± 0.10 × 10^8^ RFU (Figure 7B). This observation could be evidenced by cell-to-cell junction forming and obstructing the flow of the FITC-dextran to the lower side. Additionally, the barrier integrity of the cells after 14 and 21 days of culture on a paper membrane was investigated. As displayed in Supplementary Figure S6, at 14 days of culture, there was a 1.36-fold higher fluorescence intensity compared to the 21-day culture. This indicates that the Caco-2 cells in the 21-day culture have reached confluency and matured their tight junction proteins. This finding aligns with the ZO-1 expression observed in the immunofluorescence imaging (Figure 2B).

Interestingly, when the cells were treated overnight with NET-isolated and Staurosporine, the fluorescence intensity of 0.99 ± 0.11 × 10^8^ and 0.86 ± 0.08 × 10^8^ RFU were obtained (Figure 7B), respectively. As 1.61- and 1.39-fold intensity increased compared to the cell control experiment, it could be suggested that NET-isolated and Staurosporine could disrupt the formation of a tight junction protein and induce the loss of cell-to-cell junction, hence increasing the transportation of a fluorescence tracer to the bottom layer.

## 3. Discussion

Although the intestinal barrier function can be investigated using various methods, including TEER measurement, immunofluorescence analysis, and permeability assay, the methods are still unaffordable and limit access due to the high cost and consumable nature of commercial transwell membranes. In this work, we established an optional in vitro cell model for assessing intestinal barrier function using a low-cost customizable device for the cell-based permeability assay employing a facile, low-cost paper membrane. Utilizing our device demonstrated the evaluation of tight junction formation on a Matrigel-modified paper membrane applied with TEER measurement. As a result, TEER values were detectable in the range of 0 Ω·cm^2^ to 26 Ω·cm^2^ during 21-day culturing. Interestingly, the resistance signals are reflected in barrier integrity occurring on a paper membrane, even though this membrane exhibited a lower resistance than a 2D membrane. This result could be explained as the two-paired chopstick electrode being applied by direct current (DC) [21], which can provide a relatively low sensitivity signal. In addition, the resistance signals are directly affected by the physical properties of the membrane, such as the thickness, pore sizes, and scaffold types. However, electrochemical sensors could be used instead of TEER for an effective method to examine and measure the transepithelial resistance of intestinal barrier function on 3D scaffold membranes. Notably, electrochemical impedance spectroscopy (EIS) is one of the electrochemical sensors used to measure and monitor cell activity in several applications, including cell proliferation, cell migration, cell invasion, and cell permeability [14,36]. Furthermore, EIS can be applied with an alternative current (AC) and a wide range of frequencies that are more compatible with examining the cell activity of scaffold-based cell modeling in real time with greater sensitivity.

It should be noted that the properties of cell morphology and the characteristics of the scaffold architecture are essential factors in cell culture applications. The structural features of the scaffolds, such as pore size, microfiber alignment, surface roughness, and scaffold composition, are the parameters that can influence cell behaviors and mimic the native cell environment [20]. Previously, Whatman filter papers were used for adherence cell culture in various biological applications, for example, the study of cell invasion using Whatman grade 1 paper (pore size ~11 µm) [23] and cell permeability using Whatman CF12 filter paper [22]. In this work, we have presented a Whatman grade 602H paper (pore size < 2 µm) [37] functioning as a semipermeable membrane for the intestinal barrier model. As demonstrated by immunofluorescence images, Caco-2 cells were grown and differentiated into functional epithelial cells, highlighted by the expression of tight junction protein (ZO-1) and microvilli (villin-1), which showed a higher expression in a time-manner over the 21-day culture period. The ZO-1 protein plays an essential role in maintaining intestinal homeostasis, epithelial structure, and barrier integrity. The detection of this protein can be an indicator of epithelial cell viability and differentiation. Therefore, immunofluorescence staining of the ZO-1 protein and microvilli proteins could confirm that the paper membrane supports the desired cell phenotype. Furthermore, assessing the expression of villin-1, a brush border cytoskeletal protein localized to the apical surface of mature enterocytes, provides additional evidence of intestinal epithelial differentiation on the paper membrane [38]. In addition, SEM and immunofluorescence staining were utilized to observe the Caco-2 cell characteristics that were cultured in either a 2D or 3D system. In a 2D cell system, cells are grown on a flat surface of the PET membrane, which limits their ability to mimic the natural intestinal environment. In contrast, culturing in a 3D scaffold membrane provides structural support similar to a basement membrane, enhancing cell functionality and allowing growth in three dimensions, effectively simulating the crypt–villus structure [20,39,40]. These findings are supported by the results of immunofluorescence and SEM images, in which the cells were more physiologically shaped and likely to interact with neighboring cells by forming the cell–cell junction. Ultimately, a paper membrane provides the physical advantages of being cost-effective, simple, and offering flexible solutions. This membrane also offers biological advantages that allow the establishment of a co-culture cell model for mimicking complex tissue systems and further studying cell–cell mechanisms [41].

We further demonstrated intestinal barrier dysfunction using a biological and chemical simulator. As the first line of defense mechanism and the most abundant type of white blood cell, neutrophils can activate the process called neutrophil extracellular trap (NET). Conversely, excessive NET release can induce the breakdown of intestinal barrier function [1]. Previously, the intervention of dsDNA on the intestinal barrier of Caco-2 cells was treated with dsDNA of primary neutrophils in the range of 150 to 400 ng/mL at different incubation times [42,43,44]. The immune-mediated intestinal barrier dysfunction was demonstrated using NET-isolated supernatant from dHL-60 cells. After 6 days post-differentiation, dHL-60 cells revealed multi-lobed, banded, and segmented nuclei [45]. The NET-isolated component (dsDNA) was produced by PMA induction. Then, the quantified dsDNA was conducted to disrupt the intestinal barrier integrity overnight. Our finding showed that NET components altered the barrier integrity structure with a loss of surface microvilli. Moreover, we have demonstrated intestinal barrier dysfunction using Staurosporine, a chemical apoptosis inducer. Additionally, Staurosporine alters the tight junctional formation of Caco-2 cells and induces the apoptosis of the intestinal epithelial cells through a caspase-mediated pathway [35,46,47,48]. As a result, immunofluorescence images of Staurosporine interventions revealed the morphological change of tight junction protein and surface microvilli in a dose-dependent manner, leading to a discontinuous localization pattern at the cell boundaries and cell surfaces. This finding suggests that Staurosporine is not only able to weaken physical barriers but also impair absorptive and secretory functions. Both functions are critical to maintaining intestinal homeostasis.

FITC-dextran is a widely recognized technique for evaluating permeability within biological systems. This method utilizes fluorescent tracers of various molecular weights to effectively measure their transport through the paracellular route. Such measurements provide valuable insights into the integrity and function of the intestinal barrier, making FITC-dextran a crucial tool for assessing barrier functionality in both in vitro and in vivo experimental models [49,50,51]. In this study, we demonstrated the permeability assay employing FITC-dextran at 4 kDa of molecular weight to investigate the barrier integrity on a paper membrane under different conditions. As a result, the FITC-dextran can be passed through the thickness of a paper membrane in the condition of a cell-free membrane. Also, the different solution interventions revealed distinct levels of fluorescence intensities by measuring the culture medium from the basolateral compartment. These results suggest that our device can demonstrate the physiological conditions and investigate the intestinal barrier using an alternative method of the FITC-dextran assay. Herein, our device provides a promising platform in static conditions. An in vitro model to enhance environmental cues for the intestinal cell model was developed under fluidic conditions using a 2D rocker instrument. Simultaneously, a paper-based platform was designed with a wick-like pattern to mimic fluidic conditions. These settings could influence intervention conditions, allowing for greater diffusion and exposure to the cells [52,53]. Therefore, our device offers a simple-to-handle, low-cost, and sustainable platform for an in vitro cell model to access the intestinal barrier function, with the potential for integration into biosensor applications. Although this study introduces a valuable in vitro cell model for potential drug applications, we recognize that the proposed cell model still needs further investigation before it can be effectively applied in real-world settings. Extensive research on biological validation, including evaluations of drug absorption and comparison studies, is necessary to improve the platform’s affordability and robustness. These initiatives have the potential to greatly increase the platform’s applicability in the near future and offer a more in-depth understanding of its possible uses in drug testing and related fields.

## 4. Materials and Methods

### 4.1. Chemicals and Reagents

An alkaline phosphatase (ALP) assay kit (Colorimetric) was purchased from Abcam (Waltham, MA, USA). Fetal bovine serum (FBS), fluorescein isothiocyanate (FITC)–dextran (4 kDa), dimethyl sulfoxide (DMSO), phorbol-12-myristate-13-acetate (PMA), and Staurosporine were purchased from Sigma-Aldrich (St. Louis, MO, USA). Dulbecco’s Modified Eagle Medium (DMEM), Roswell Park Memorial Institute (RPMI) 1640 medium with and without phenol red, and Whatman 602H grade filter paper were purchased from Cytiva (Marlborough, MA, USA). Matrigel^®^ was purchased from Corning (New York, NY, USA). Hoechst 33342 was purchased from Dojindo Laboratories (Kumamoto, Japan). Polylactic acid (PLA) filament was purchased from Polymaker (Shanghai, China). Rabbit monoclonal Anti-ZO-1, Mouse monoclonal Anti-Villin-1, Alexa Fluor 488-conjugate Anti-rabbit IgG, Alexa Fluor 594-conjugate Anti-mouse IgG antibodies, and PI staining solution were purchased from Cell Signaling Technology (Danvers, MA, USA). Trypan blue solution, Calcein-AM, and Quant-iT^TM^ PicoGreen^TM^ dsDNA assay kits were purchased from Thermo Fisher Scientific (Waltham, MA, USA).

### 4.2. Cell Culture and Differentiation

The Caco-2 cell line is isolated from colon tissue derived from a 72-year-old white male with colorectal adenocarcinoma, one of the most common cell lines used to study intestinal barrier function [9]. Caco-2 cells were purchased from the American Type Culture Collection (ATCC). Cells were cultured in DMEM medium supplemented with 10% (*v*/*v*) FBS, antibiotic-antimycotic solution, and grown in 5% CO_2_ at 37 °C. Caco-2 cells were seeded at an initial cell density of 1 × 10^5^ and 5.0 × 10^5^ cells/cm^2^ on a paper membrane of a 3D-printed transwell device to establish a paper-based intestinal barrier. Then, cells were cultured for differentiation and polarization for 14–21 days to reach the confluent monolayer formation [6].

In this work, we proposed the HL-60 cell line as a neutrophil cell model for neutrophil extracellular trap (NET) formation. HL-60 is the human leukemia cell line isolated from the peripheral blood derived from a 36-year-old Caucasian female with acute promyelocytic leukemia. HL-60 cells were purchased from the American Type Culture Collection (ATCC). The cells were cultured in RPMI 1640 medium supplemented with 10% (*v*/*v*) FBS, HEPES, antibiotic antimycotic solution and grown in 5% CO_2_ at 37 °C. Cells were passaged no longer than 15 times [54,55]. HL-60 cells were induced to neutrophil-like cells or dHL-60 cells by incubating with 1.3% DMSO for 6 days [54,56,57]. After incubation, the cell viability was assessed by a trypan blue exclusion assay. Furthermore, we investigated the nuclear morphology of dHL-60 cells by Hoechst 33342 staining, a nuclear indicator [56]. Moreover, both undifferentiated and differentiated cells observed the morphological change under a light microscope on day 1 and day 6.

### 4.3. Transwell Device and Paper Membrane Preparation

A fused filament fabrication (FFF) 3D printer (i3Dbot x250 3D printer, Bangkok, Thailand) was employed to create the 3D-printed transwell device using PLA filament. Additionally, a Whatman cellulose filter paper (602H grade) (Cytiva, MA, USA) with a pore size of less than 2 μm and a culturing area diameter of 7.5 mm was used as the semipermeable membrane for the transwell device. All design files were used following the previously described method [26]. Briefly, a wax-pattern paper was printed by a wax printer (ColorQube 8570, Xerox Corporation, Norwalk, CT, USA). Then, the paper was heated on a hot plate at 150 °C for 2 min to melt the wax into the microfiber of the paper [36]. The paper membrane was coated with 10 µL of Matrigel^®^ solution (11 mg/mL) on a 0.44 cm^2^ growth area, constructing an appropriate microenvironment for cell growth. Subsequently, the paper membrane was incubated for 30 min at 37 °C in a 5% CO_2_ environment to promote the formation of a basement membrane [58].

### 4.4. TEER Measurements

In this work, we aimed to measure the transepithelial electrical resistance (TEER) of intestinal barrier formation on a paper membrane using a Millicell^®^ ERS-2 voltohmmeter (Merck, MERS00002, Rahway, NJ, USA) with a chopstick electrode (Merck, MERSSTX01). The experiment was performed on a polyethylene terephthalate (PET) membrane (6.5 mm diameter and 0.4 µm pore size) of Millicell^®^ Cell Culture Inserts (Merck, PTHT24H48). On the day of measurement, the cells were replaced with a fresh culture medium, and the device was kept at room temperature for 5 min to allow for temperature equilibration. After that, the resistance signal was measured from three different corners of the paper membrane. Additionally, a cell-free paper membrane was used as a blank. The calculation was followed by the previous equation [59]. The TEER value (Ω × cm^2^) was calculated by subtracting the blank resistance (R_blank_) from the resistance across the sample (R_sample_) and then multiplying it with the growth area (A) of the membrane insert.

TEER values (Ω × cm^2^) = (R_sample_ − R_blank_) (Ω) × Area (cm^2^)

### 4.5. Alkaline Phosphatase (ALP) Activity Assessment

The alkaline phosphatase (ALP) activities of Caco-2 cells (1.0 × 10^5^ cells/cm^2^) in both 2D and 3D membranes were quantified according to the manufacturer’s protocol. Briefly, the culture medium of the apical compartment was collected at different time points. The samples were incubated with the substrate of 5 mM *p*-nitrophenyl phosphate (PNPP) solution at 25 °C for 60 min, during which the enzyme will convert the PNPP substrate to colored *p*-nitrophenol (PNP). After that, the stop solution was added, and the samples were determined at 405 nm using a SpectraMax iD3 Multi-Mode microplate reader (Molecular Devices, San Jose, CA, USA).

### 4.6. NET Formation

In this experiment, neutrophil extracellular trap (NET) formation was stimulated using PMA induction. PMA is a free molecule that can cross the plasma membrane and directly activate protein kinase C by promoting NOX2 activation [60]. For NET formation, dHL-60 cells were incubated with PMA (100 nM) for 4 h.

Next, we quantified the dsDNA generated by neutrophils using PicoGreen^®^ dsDNA Quantitation Reagent. The samples were centrifuged at 200× *g* at 4 °C for 5 min. After separating the supernatant, the pellet was resuspended in PBS. The supernatants were collected and stored at −80 °C until use in future experiments, following the method described previously [55,61]. The measurement of dsDNA followed the manufacturer’s instructions and was performed using SpectraMax iD3 Multi-Mode Microplate Reader (Molecular Devices, San Jose, CA, USA) at a wavelength of 480 nm/520 nm (excitation/emission).

### 4.7. Interventions for Intestinal Barrier Dysfunction

In this work, intestinal barrier dysfunction of Caco-2 cells cultured on a paper membrane was assessed using biological and chemical stimulators. For the experimental conditions, devices were divided into two groups, including (i) in cell control groups, the cells were exposed to the culture medium only; (ii) in cell-treated groups, cells were exposed to the culture medium containing either NET-dsDNA (20 and 200 ng/mL dsDNA) or Staurosporine (1 and 10 µM) then incubate at 37 °C overnight. After the intervention, a paper membrane was harvested and investigated using immunofluorescence analysis.

### 4.8. Fluorescence and Immunofluorescence Analysis

The differentiation of HL-60 cells and the NET formation of PMA-induced dHL-60 cells was performed using Hoechst 33342 staining, a nuclear indicator. Cells were gently washed with PBS, and then stained cells were observed under a confocal laser scanning microscope (ZEISS LSM 980, Oberkochen, Germany).

In the preparation of immunofluorescence staining, the cells were washed twice with PBS, then fixed with 4% paraformaldehyde (PFA) phosphate buffer solution for 20 min at room temperature (RT). After that, the fixed cells were washed with PBS, immediately permeabilized with 0.5% Triton X-100, and incubated at RT for 30 min. Then, cells were washed with PBS and blocked with bovine serum albumin (2% BSA in PBS) for 90 min at RT. For the incubation of primary antibodies, the cells were incubated with rabbit monoclonal Anti-ZO-1 (1:200) and mouse monoclonal Anti-Villin-1 (1:200) overnight at 4 °C. After completing the primary incubation, the cells were washed with PBS and incubated with secondary antibodies of Alexa Fluor 488-conjugate Anti-rabbit IgG (1:2000) and Alexa Fluor 594-conjugate Anti-mouse IgG (1:2000) antibodies for 1 h. Lastly, the cells were washed and immediately incubated with Hoechst 33342 (10 µM) solution at RT for 1 h. The stained cells were investigated under a confocal laser scanning microscope.

The viability of cells cultured on a paper membrane was performed using LIVE/DEAD staining dye. Caco-2 cells that had been cultured for 21 days were incubated overnight under different conditions. Next, the cells were washed and stained with 4 µg/mL of Calcein-AM solution and 10 µg/mL of propidium iodide (PI) solution at 37 °C, 5% CO_2_ incubator for 20 min. After that, the stained cells were gently washed with PBS before being placed on a glass slide and then immediately performed for fluorescence imaging using a BioTek Cytation 7 cell imaging multimode reader (Agilent, Santa Clara, CA, USA).

### 4.9. Permeability Assay

A paper-based intestinal barrier permeability assay was performed using FITC-Dextran (4 kDa) permeability assay. After cultured Caco-2 cells on a Matrigel-functionalized membrane, the apical compartment was replaced with 300 µL of different conditions; for cell-free and cell control were exposed to the phenol red-free culture medium only, while the intervention of NET and Staurosporine conditions, cells were exposed to the solution of NET-isolated and Staurosporine, respectively. All conditions were also suspended with FITC-dextran dye (5 mg/mL), and then cells were incubated overnight in a 37 °C incubator. In the measurement of fluorescence intensity, the culture medium was collected from a basolateral compartment and transferred to a 96-well black flatted-bottom plate. Additionally, the solution of phenol red-free medium was used as a blank. The fluorescence intensity was measured at the wavelength of excitation 490 and emission 530 nm using SpectraMax iD3 Multi-Mode Microplate Reader (Molecular Devices, San Jose, CA, USA).

### 4.10. Scanning Electron Microscopy (SEM)

Cell surface membrane characteristics were observed at different zones, including wax-printed, plain, and Matrigel-modified areas. Furthermore, Caco-2 cells were cultured on both 2D and 3D paper-based scaffold membranes to observe the physical morphology on 21-day cultures. Cell membranes were fixed and preserved with 2.5% glutaraldehyde in cold PBS. Then, the sample was gradually dehydrated by increasing ethanol concentrations in distilled water (30%, 50%, 70%, 90%, and 100%) for 15 min each. Lastly, the sample was sputter-coated with platinum and then examined with scanning electron microscopy (ESEM) (Quattro ESEM, Thermo Scientific™, Waltham, MA, USA), which was performed by the NSTDA Characterization and Testing Service Center (NCTC), Pathum Thani, Thailand.

### 4.11. Statistical Analysis

Statistical analysis was conducted using GraphPad Prism Version 10.1.1. Data are presented as mean ± standard deviation (SD). A *p*-value less than 0.05 was considered statistically significant. The ALP activity measurement was evaluated using two-way ANOVA followed by Tukey’s multiple comparison test.

## 5. Conclusions

This study presented a promising in vitro method for intestinal barrier function employed with a 3D-printed transwell device and a paper membrane insert. Our device offers a low-cost, sustainable, and cost-effective solution for the transwell-based assay. Furthermore, a Matrigel-functionalized paper membrane offers a suitable 3D microenvironment for cell growth, proliferation, and differentiation of Caco-2 cells, suggesting that it can be used to mimic the basement membrane of intestinal barrier function, and its future use may be possible to establish the co-culture system by the combination of various types of cells cultured on different membranes. Moreover, this device showed the potential for measuring the tight junction formation by employing a chopstick electrode of TEER measurements and immunofluorescence staining. These results suggest that it can be integrated with electrochemical sensors for real-time monitoring, which can spur various applications such as understanding biological mechanisms, drug screening, and drug discovery.

## Data Availability

Data are contained within the article or Supplementary Materials.

## Supplementary Materials

The supporting information can be downloaded at https://www.mdpi.com/article/10.3390/ijms26062524/s1. References [42,43,44,62,63] are cited in the Supplementary Materials.
