# Supplementary material for: An In Vitro Cell Model of Intestinal Barrier Function Using a Low-Cost 3D-Printed Transwell Device and Paper-Based Cell Membrane
Source: Int J Mol Sci. 2025 Mar 12;26(6):2524. doi: 10.3390/ijms26062524 (PMC11941856; doi:10.3390/ijms26062524)
Supplement: Supplementary file 1 [file ijms-26-02524-s001.zip › ijms-3429907-supplementary.pdf]

Supplementary data

An In Vitro Cell Model of Intestinal Barrier Function Using a Low-Cost 3D-Printed Transwell Device and Paper-Based Cell Membrane

Pitaksit Supjaroen [1], Wisanu Niamsi [1], Parichut Thummarati [2,3] and Wanida Laiwattanapaisal [2,3,*]

[1] Graduate Program in Clinical Biochemistry and Molecular Medicine, Department of Clinical Chemistry, Faculty of Allied Health Sciences, Chulalongkorn University, Bangkok 10330, Thailand; pitaksit.sa@gmail.com (P.S.); 6671003637@student.chula.ac.th (W.N.)
[2] Centre of Excellence for Biosensors and Bioengineering (CEBB), Department of Clinical Chemistry, Faculty of Allied Health Sciences, Chulalongkorn University, Bangkok 10330, Thailand; parichut.t@chula.ac.th
[3] Department of Clinical Chemistry, Faculty of Allied Health Sciences, Chulalongkorn University, Bangkok 10330, Thailand
* Correspondence: wanida.l@chula.ac.th

1. TEER measurement of a commercial 2D transwell membrane insert

Caco-2 cells were seeded on a 24-well plate polyester (PET) membrane insert at different densities including 3.0×10^4, 6.0×10^4, 8.5×10^4, and 1.0×10^5 cells/cm^2 on a polyethylene Terephthalate (PET) membrane (6.5 mm diameter and 0.4 µm pore size) of Millicell™ hanging cell culture insert (Merck, PTHT24H48). The TEER measurement was performed every second day for 21 days [1].

The TEER measurement of the Caco-2 cell cultured on a 2D cell culture membrane insert among four different densities is shown in Supplementary Figure S1B. The cell density at 1.0×10^5 cells/cm^2 exhibited the highest magnitude (293 ± 2 Ω•cm^2) at the 15-day culture, suggesting that the cells reached their confluency and the tight junction maturation on the 2D surface membrane. Moreover, the TEER values were continuously decreased until the 21-day culture. This finding suggests that the 2D membrane is a system that provides a limited growth area of Caco-2 cell culture.

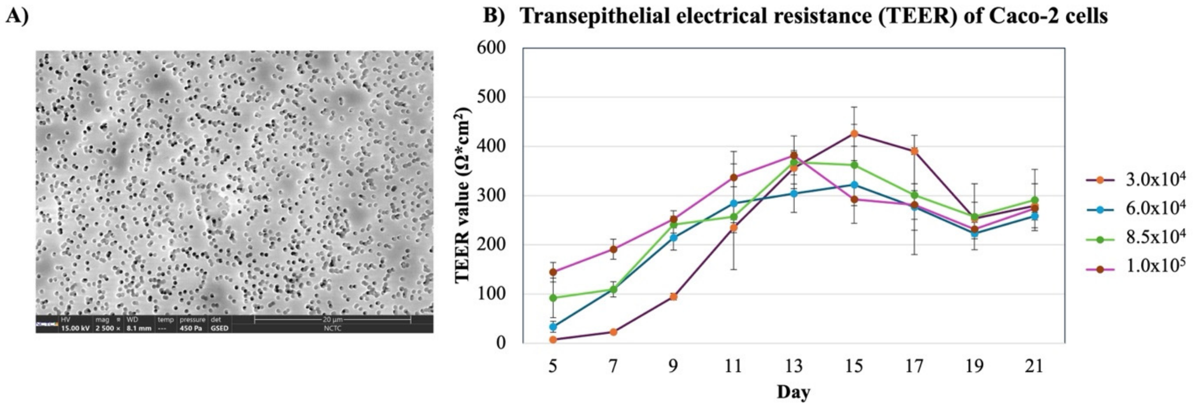

Supplementary Figure S1 (A) The ESEM image of commercial 2D transwell membrane insert. (B) TEER measurement of a 2D transwell membrane inserts at different initial densities.

2. NET formation of HL-60 cell line

2.1. DMSO-dHL-60 cell differentiation

The observation of the physical morphology and morphological change of HL-60 cells was performed under a light microscope on day 1 and day 6 during the differentiation processes as shown in Supplementary Figure S2.

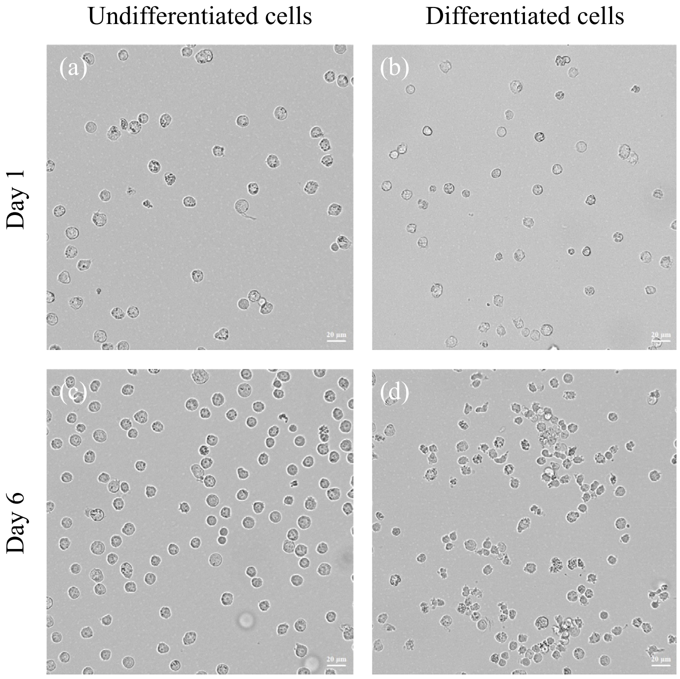

Supplementary Figure S2 Light microscopic observation of undifferentiated HL-60 cells and differentiated HL-60 cells at day 1 (A, B) and day 6 (C, D). The scale bar is 20 µm.

2.2. Cell viability using trypan blue exclusion assay

The assessment of the cell viability of dHL-60 cells post-differentiation was performed using a trypan blue exclusion assay. The results showed good viability of HL-60 cells (undifferentiated cells) and dHL-60 cells (differentiated cells) with 97.54±0.39% and 85.98±1.50%, respectively, as presented in Supplementary Figure S3. However, DMSO does not affect cell viability but it reduces cell proliferation during the differentiation process [2].

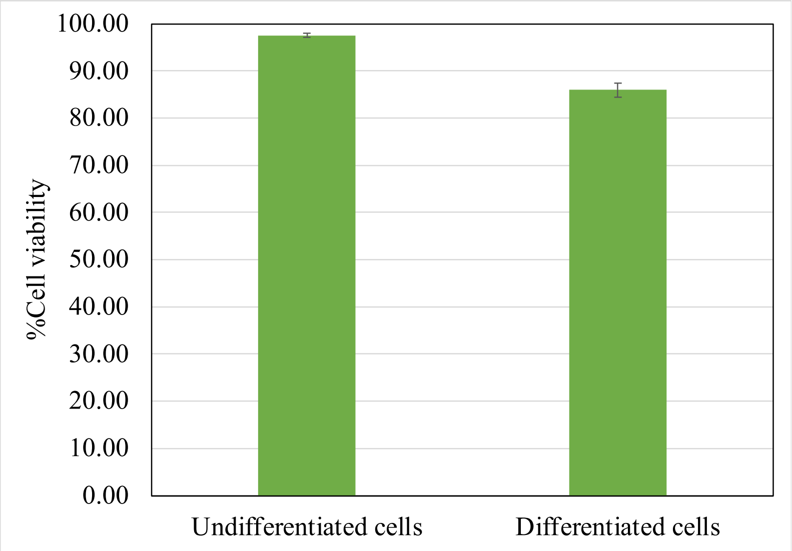

Supplementary Figure S3 The percentage of HL-60 and DMSO-dHL-60 cell viability on days 6, post-differentiation using trypan blue exclusion assay.

2.3. NET quantification

In this study, HL-60 cells were used to present a primary neutrophil cell model. For the NET formation, the experimental steps were performed by following previous publications with some modifications of seeding density, buffer solution, and washing steps. Finally, the dsDNA was collected and stored at -80°C until use.

To determine the dsDNA levels of PMA-induced dHL-60 cells using the PicoGreen® dsDNA Quantitation Reagent. The NET-dsDNA was activated through PMA-induced dHL-60 cells from two different cell densities. Following the previous publication's protocol, the dHL-60 cells released the dsDNA in a range of primary neutrophils that can produce the dsDNA at 150-400 ng/ml [3-5]. Subsequently, the dsDNA contents were further adjusted to the desired concentrations (20 and 200 ng/ml) for the demonstration of biological-induced intestinal barrier dysfunction.

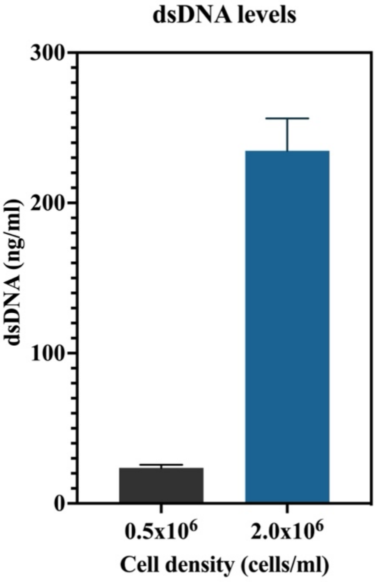

Supplementary Figure S4 The quantification of NET-dsDNA from PMA-induced dHL-60 cells using PicoGreen® dsDNA Quantification reagent.

3. LIVE/DEAD staining

The assessment of cell viability relates to the formation of the ZO-1 protein. Even, the cells were lost of tight junction protein that leads to increased PI uptake, indicating tight junction dysfunction. As demonstrated by Supplementary Fig. S5, the green fluorescence signal of Calcein-AM represents an intracellular esterase activity of viable cells. In contrast, the red fluorescence signal represents the binding of PI fluorescence dye to the DNA of dead cells. As a result, the NET and Staurosporine stimulations revealed an intense red fluorescence signal of dead cells while the control of cells exhibited a low signal of dead cells, highlighting these stimulations reduce the cell viability through their mechanism and lead to the loss of cell barrier integrity.

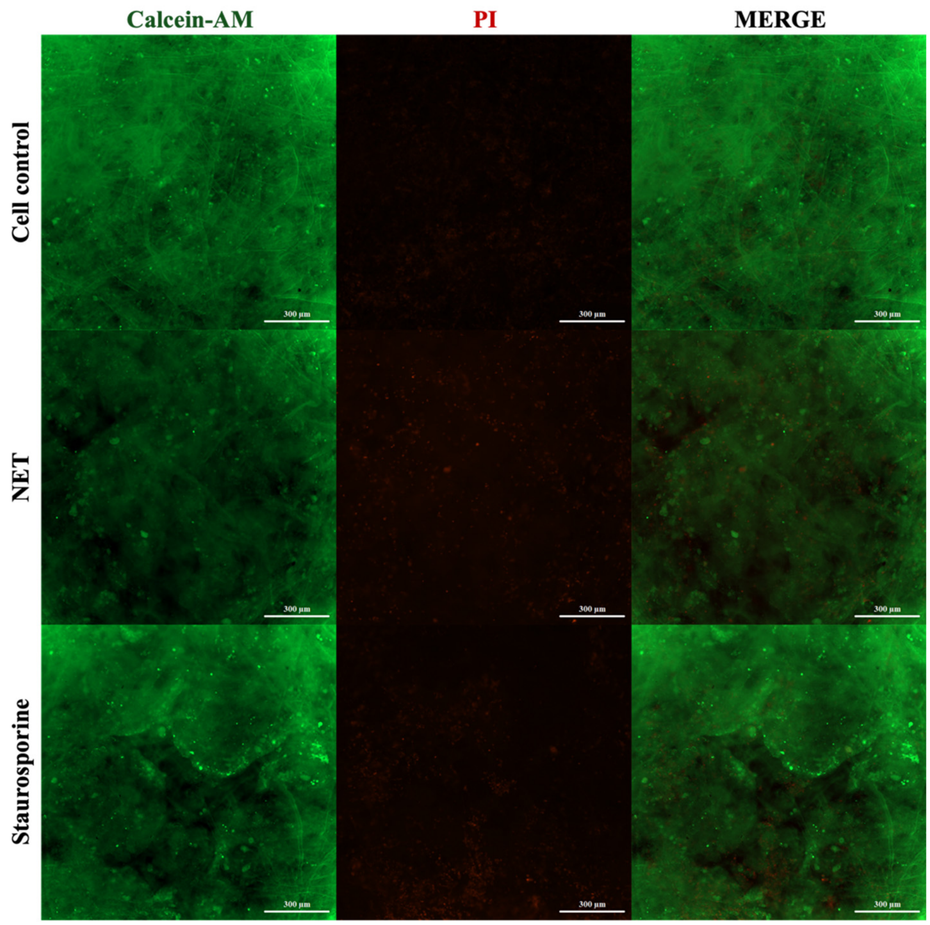

Supplementary Figure S5 Representative fluorescence images of intestinal epithelial cells after incubating with different conditions using LIVE/DEAD staining.

4. **Permeability assay**

Following 14 and 21 days cultured on a paper membrane, the permeability assay was performed to evaluate the intestinal barrier integrity using FITC-dextran (4 kDa). As a result, the fluorescence intensity of cell-free membranes revealed a similar level each day. Interestingly, the 14-day culture of Caco-2 cells exhibited a 1.36-fold higher fluorescence intensity than the 21-day culture, suggesting the intestinal barrier integrity over time. This finding demonstrates that FITC-dextran offers an alternative method to evaluate the barrier integrity on a paper membrane whether TEER measurement presents a low magnitude of the intestinal barrier function.

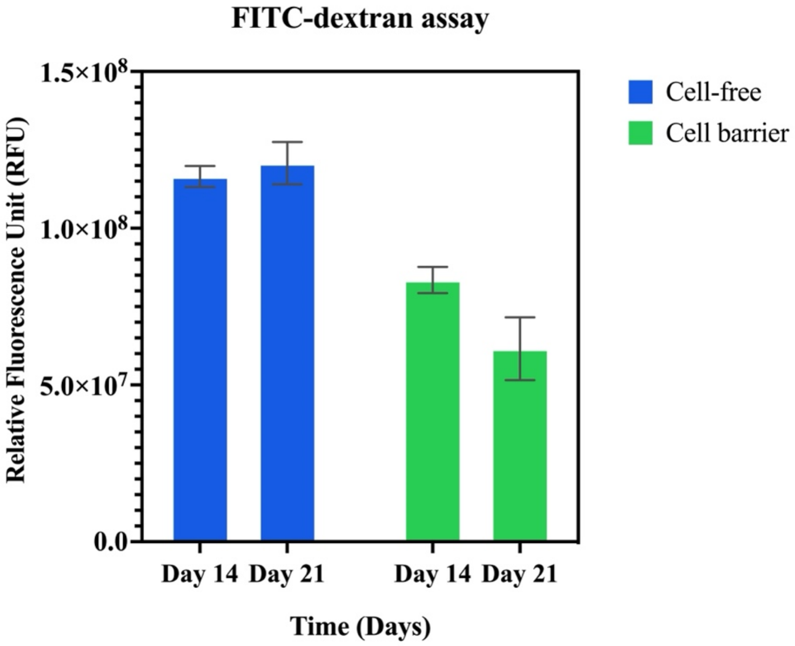

Supplementary Figure S6 Permeability assay of 14- and 21-day cultured Caco-2 cells on a paper membrane.

References

1. Hesler, M.; Schwarz, D.H.; Dähnhardt-Pfeiffer, S.; Wagner, S.; von Briesen, H.; Wenz, G.; Kohl, Y. Synthesis and in vitro evaluation of cyclodextrin hyaluronic acid conjugates as a new candidate for intestinal drug carrier for steroid hormones. *European Journal of Pharmaceutical Sciences* **2020**, *143*, 105181, doi:https://doi.org/10.1016/j.ejps.2019.105181.
2. Zhao, Y.; Zhang, Y.; Liang, X.; Liu, S.; Cao, X.; Chen, N.; Chen, Z.; Yan, J. Monitoring the differentiation of dimethyl sulfoxide-induced human leukemia (HL-60) cells by Raman spectroscopy. *Journal of Raman Spectroscopy* **2021**, *52*, 1086-1094, doi:https://doi.org/10.1002/jrs.6122.
3. Lai, H.J.; Doan, H.T.; Lin, E.Y.; Chiu, Y.L.; Cheng, Y.K.; Lin, Y.H.; Chiang, H.S. Histones of Neutrophil Extracellular Traps Directly Disrupt the Permeability and Integrity of the Intestinal Epithelial Barrier. *Inflamm Bowel Dis* **2023**, *29*, 783-797, doi:10.1093/ibd/izac256.
4. Sun, S.; Duan, Z.; Wang, X.; Chu, C.; Yang, C.; Chen, F.; Wang, D.; Wang, C.; Li, Q.; Ding, W. Neutrophil extracellular traps impair intestinal barrier functions in sepsis by regulating TLR9-mediated endoplasmic reticulum stress pathway. *Cell Death & Disease* **2021**, *12*, 606, doi:10.1038/s41419-021-03896-1.
5. Chu, C.; Wang, X.; Chen, F.; Yang, C.; Shi, L.; Xu, W.; Wang, K.; Liu, B.; Wang, C.; Sun, D.; et al. Neutrophil extracellular traps aggravate intestinal epithelial necroptosis in ischaemia-reperfusion by regulating TLR4/RIPK3/FUNDC1-required mitophagy. *Cell Prolif* **2024**, *57*, e13538, doi:10.1111/cpr.13538.
